# Relationship Between Cervical Central Canal and Neural Foraminal Dimensions in a Normative Population

**DOI:** 10.3390/tomography12060086

**Published:** 2026-06-12

**Authors:** Kai Nguyen, Zachary Brandt, David Shin, Carson Cummings, Rohan Kubba, Jacob Razzouk, Davis Carter, Mei Carter, Wayne Cheng, Olumide Danisa

**Affiliations:** 1 Department of Orthopaedic Surgery, Loma Linda University Health, Loma Linda, CA 92354, USA; 2 School of Medicine, Loma Linda University, Loma Linda, CA 92350, USA; 3 College of Medicine, California Northstate University, Elk Grove, CA 95757, USA; rohan.kubba12079@cnsu.edu; 4 Department of Anesthesiology, Loma Linda University Health, Loma Linda, CA 92354, USA; 5 Department of Orthopaedic Surgery, Jerry L. Pettis Veterans Affairs (VA) Medical Center, Loma Linda, CA 92357, USA; 6 Department of Orthopaedic Surgery and Neurosurgery, Duke University Medical Center, Durham, NC 27710, USA

**Keywords:** cervical spine, spinal canal, neural foramen, morphometry, computed tomography, normative anatomy

## Abstract

Background/Objectives: The relationship between cervical central canal and neural foraminal dimensions remains undefined. This study examined correlations between these structures in a young adult CT cohort screened to exclude apparent cervical spinal pathology. Methods: We retrospectively reviewed computed tomography images of 1000 patients aged 18 to 35 years screened to exclude apparent cervical spinal pathology. Central canal dimensions included anteroposterior diameter, interpedicular distance, and cross-sectional area. Neural foraminal dimensions included axial width, craniocaudal height, and area. Pearson correlation tests were used to assess associations between the central canal and neural foraminal dimensions. Results: Neural foraminal area showed the most consistent associations with interpedicular distance bilaterally, though these relationships were modest-to-moderate in magnitude. Axial width and craniocaudal height exhibited no consistent correlations with central canal dimensions. No strong correlations were observed between any combination of central canal and neural foraminal dimensions at any disc level. Conclusions: In this young adult CT cohort without apparent cervical spinal pathology, cervical central canal and neural foraminal dimensions demonstrated no strong correlations across levels. These findings suggest that central canal dimensions should not be used as a surrogate for neural foraminal dimensions in quantitative morphometric assessment.

## Simple Summary

The relationship between the size of the cervical spinal canal and the neural foramina is not well understood, despite both being important in the evaluation of cervical stenosis. In this study, we analyzed CT imaging from 1000 young adult patients who were screened to exclude apparent cervical spinal pathology. We found no strong correlations between central canal and neural foraminal measurements. The neural foraminal area showed modest-to-moderate associations with interpedicular distance, while most other relationships were weak or inconsistent. These findings suggest that central canal dimensions should not be used as a surrogate for foraminal dimensions in quantitative morphometric assessment and provide a baseline for future studies in symptomatic or degenerative populations.

## 1. Introduction

Normative cervical spinal anatomy has been increasingly characterized through cross-sectional imaging. Within the cervical spine, two key anatomic structures implicated in stenosis are the central spinal canal and neural foramen. The size of the central spinal canal has been explored in prior studies and is generally well agreed upon [1,2,3]. More recently, normative values for cervical neural foraminal dimensions (NFD) have been described, for which there has historically been little consensus [4].

Although cervical central canal and neural foraminal dimensions have each been independently characterized, no study has directly evaluated whether these structures correlate in size within the same patients, across multiple cervical levels, in a large CT cohort. Cervical central canal stenosis and neural foraminal stenosis are clinically distinct but anatomically adjacent processes. Central canal narrowing is most closely associated with cervical myelopathy, whereas foraminal narrowing is more closely associated with cervical radiculopathy. Although both compartments are routinely evaluated together on cervical imaging, quantitative morphometric studies have generally assessed them separately. In the lumbar spine, a prior study demonstrated a significant correlation between the sagittal diameter of the spinal canal and the sagittal dimensions of the intervertebral foramen, concluding that patients with developmental central stenosis likely harbor concomitant foraminal stenosis [5]. Whether a comparable relationship exists in the cervical spine has not been investigated. This question also has relevance for future surgical and degenerative disease studies, as some cervical procedures can affect both the central canal and neural foramina. Understanding whether these dimensions correlate at baseline may help frame future work evaluating how each compartment changes after intervention.

This study evaluates the correlation between cervical central canal and neural foraminal dimensions using computed tomography (CT) of young adult patients screened to exclude apparent cervical spinal pathology. Using a cohort without apparent cervical spinal pathology, we aimed to characterize the relationship between cervical central canal and neural foraminal dimensions within the same individuals across multiple levels in a large young adult CT cohort. We hypothesized that these dimensions would demonstrate positive but limited correlations, reflecting shared vertebral anatomy but distinct compartment-specific morphology.

## 2. Materials and Methods

Following IRB approval (#5240118; approved 18 March 2024), we retrospectively reviewed cervical spine CTs without contrast and soft tissue neck CTs performed at our institution between January 2014 and January 2023 using a Discovery CT750 HD 64-slice CT scanner (GE Healthcare, Waukesha, WI, USA). Imaging was considered eligible for opportunistic morphometric assessment when review of the radiographic report and electronic medical record did not identify known cervical spinal pathology or exclusionary symptoms. Patients were reviewed in a systematic, consecutive manner until a sample size of 1000 eligible patients was reached. Patients between 18 and 35 years of age were included. Exclusion criteria included congenital cervical stenosis, achondroplasia, cerebral palsy, Klippel–Feil anomaly, documented neck or upper-extremity pain, upper-extremity numbness, traumatic spinal injury with or without osseous injury, spinal malignancy, spinal infection, existing spinal hardware, or prior cervical spine surgery. Relevant spinal pathology and symptom history were identified through review of radiographic reports and the electronic medical record. A patient was identified as having congenital cervical stenosis if any C3–C7 interpedicular distances were smaller than previously published threshold values [6]. Due to the retrospective, radiographic nature of the study, patient consent was not required. Patient age, height, weight, body mass index (BMI), sex, race, and ethnicity were recorded at the time of imaging; race and ethnicity were obtained from self-reported entries in the electronic medical record.

**Figure 1:**
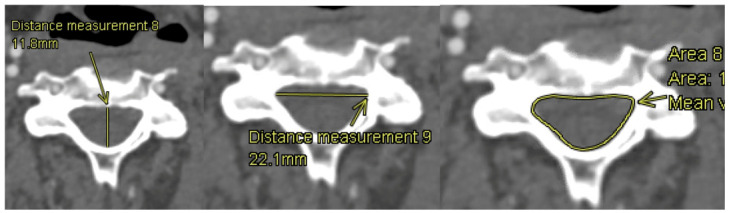
Cervical central canal dimension measurements, shown from left to right: anteroposterior (AP) diameter, interpedicular distance (IPD), and cross-sectional area.

Images were reviewed using the IMPAX6 picture archiving and communication system (Agfa-Gevaert, Mortsel, Belgium) with window and level designations of 2000 Hounsfield units (HU) and 500 HU, respectively. Coronal, sagittal, and axial multiplanar reconstructions were utilized for measurement and visual reference. Measurement definitions were based on established cervical morphometric methods [1,7,8]. Medical students were trained by a board-certified neuroradiologist to measure the dimensions of the cervical central canal, defined as follows: anteroposterior (AP) diameter, interpedicular distance (IPD), and cross-sectional area. Anteroposterior (AP) diameter was defined as the distance from the posterior cortex of the vertebral body to the anterior osseous boundary of the posterior canal on axial imaging. Interpedicular distance (IPD) was defined as the longest transverse distance between the medial borders of the left and right pedicles on axial imaging. The cross-sectional area was calculated according to the borders of the central canal bony outline using the IMPAX6 tracing freeform tool in the axial view. Figure 1 provides an illustration of the measurement technique for all central canal dimensions.

**Figure 2:**
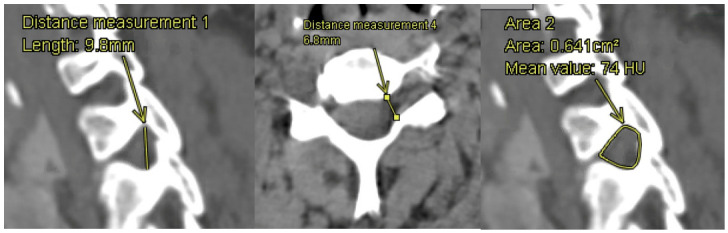
Cervical neural foraminal dimension measurements, shown from left to right: craniocaudal height, axial width, and foraminal area.

Medical students were also trained to measure cervical neural foraminal dimensions (NFD), defined as follows: axial width, craniocaudal height, and area. Neural foraminal width was defined as the shortest distance between the posterior-inferior corner of the superior vertebra and the anterior border of the superior articular process of the lower vertebra as measured on the axial view. Foraminal height was defined as the longest distance between the borders of the upper and lower pedicles viewed in the sagittal view. The neural foraminal area was measured by manually tracing the bony foraminal boundary on the parasagittal image using the IMPAX6 freeform tracing tool. The traced boundary was defined by the posterior vertebral body cortex and disc anteriorly, the superior and inferior pedicle cortices cranially and caudally, and the superior articular process of the inferior vertebra posteriorly. Figure 2 provides an illustration of the measurement technique for all NFDs.

All measurements were obtained at each intervertebral disc level from C2–C3 to C7–T1. Left and right foraminal measurements were analyzed separately to preserve anatomic variability. Measurements were obtained on the axial or sagittal image that best demonstrated the relevant osseous landmarks for each parameter. Interobserver reliability was assessed by duplicate measurements of the first 200 subjects by two independent reviewers using the intraclass correlation coefficient (ICC) two-way mixed model on absolute agreement. ICC was categorized as poor, fair, good, or excellent based on thresholds of <0.40, 0.40–0.59, 0.60–0.74, and >0.75, respectively. The overall ICC was 0.792 (95% CI, 0.736–0.842), indicating good-to-excellent reliability. The remaining measurements were performed by one reviewer per CT scan [9].

Statistical analyses were performed using SPSS version 28 (IBM Corporation, Armonk, NY, USA), with alpha set at 0.05. Kolmogorov–Smirnov tests and Q-Q plots were used to assess data normality, and Levene’s test and regression residual plots were used to evaluate homoscedasticity. Pearson correlation tests were then performed to assess bivariate associations between each central canal dimension (AP diameter, IPD, cross-sectional area) and each neural foraminal dimension (axial width, craniocaudal height, area) at each disc level from C2–C3 to C7–T1, for both left and right sides. Correlation coefficients were categorized as weak (r = 0–0.39), moderate (r = 0.40–0.69), or strong (r ≥ 0.70) [10]. Given the large sample size, statistically significant *p*-values may occur even for modest correlations; interpretation therefore emphasized correlation magnitude and consistency of patterns across cervical levels rather than *p*-values alone. *p*-values were not adjusted for multiple comparisons; emphasis was placed on correlation magnitude and consistency of patterns across levels. Descriptive statistics consisted of means, standard deviations (SD), ranges, and 95% confidence intervals (CI).

## 3. Results

### 3.1. Cohort Description

A total of 4762 subjects were screened, of which 3762 were excluded. Of those excluded, 1309 were outside the 18–35-year age range, 967 had incomplete or poor-quality imaging, 486 had a history of prior spinal surgery, 354 had documented neck or upper-extremity pain or numbness, 327 had congenital cervical stenosis, 264 had traumatic spinal injury, 27 had spinal infection, 22 had malignancy, 4 had cerebral palsy, and 2 had achondroplasia. Of the 1000 patients included, 486 were female and 514 were male. Mean age was 26.1 ± 5.9 years, and mean BMI was 27.9 ± 8.0 kg/m^2^. With respect to race and ethnicity, 357 (35.7%) were Hispanic, 245 (24.5%) White, 144 (14.4%) Black, 83 (8.3%) Asian, 18 (1.8%) other, and 153 (15.3%) unavailable.

### 3.2. Cervical Central Canal and Neural Foraminal Dimensions

Mean cervical central canal and neural foraminal dimensions at each disc level are presented in Table 1.

### 3.3. Correlation Between Cervical Central Canal and Neural Foraminal Dimensions

Pearson correlations between cervical central canal dimensions and neural foraminal area at each disc level are presented in Table 2.

Across C2–C3 through C6–C7, the neural foraminal area showed the most consistent associations with central canal dimensions, particularly IPD. These IPD-foraminal area associations were modest-to-moderate in magnitude (left: r = 0.504–0.602; right: r = 0.535–0.602). These associations remained limited in explanatory value, as even the highest correlations were below the threshold for a strong relationship. Correlations between foraminal area and AP diameter or central canal cross-sectional area were generally weak (r < 0.40). Foraminal axial width and craniocaudal height did not demonstrate consistent associations with any central canal dimension at any level. No canal-to-foramen correlation reached r ≥ 0.70 at any disc level.

At C7–T1, IPD measurements were not available, limiting direct comparison with the C2–C3 through C6–C7 findings; results at this level should be interpreted accordingly. Complete Pearson correlation coefficients for all central canal and neural foraminal dimension pairings at each disc level are provided in Supplementary Table S1.

## 4. Discussion

This study found limited correlation between cervical central canal and neural foraminal dimensions in a young adult CT cohort without apparent cervical spinal pathology. Across disc levels, the most consistent relationship was between interpedicular distance and neural foraminal area, though this association was only modest-to-moderate in strength. Correlations involving anteroposterior diameter and cross-sectional area were weak, and no strong correlations were observed at any level. These findings indicate that cervical central canal dimensions do not reliably predict neural foraminal dimensions in this cohort, and that quantitative assessment of each compartment is necessary for accurate morphometric characterization.

Prior studies have independently characterized cervical central canal and neural foraminal anatomy. Other studies have established normative central canal dimensions [1,2,3], while more recent work has described neural foraminal measurements in a large CT-based cohort [4]. Similar morphometric correlation approaches have been applied across spinal regions [11,12]. However, the dimensional relationship between these structures has not been directly evaluated. The present study addresses this gap by correlating central canal and foraminal measurements within the same young adult CT cohort, providing a more integrated understanding of cervical spinal morphology.

Among the central canal parameters, IPD showed the most consistent association with neural foraminal area, a finding that likely reflects shared dependence on transverse vertebral anatomy. The pedicles form both the lateral boundaries of the central canal and the superior and inferior borders of the neural foramen, meaning that variations in transverse vertebral development may influence both measurements simultaneously [7,13,14]. A proposed right triangle model of cervical spinal anatomy suggests that central canal dimensions are shaped by interacting posterior element components rather than a single linear measurement [15]. Our findings suggest that IPD may additionally be implicated in the relationship between the central canal and neural foramina. However, the foraminal area is also shaped by several additional structures, including the facet joints, posterolateral vertebral body, uncovertebral region, and intervertebral disc height, which are not captured by IPD alone. This may explain why the association was present but not strong.

These findings contrast with observations in the lumbar spine. A prior lumbar morphometric study demonstrated a significant correlation between the sagittal diameter of the lumbar spinal canal and the sagittal dimensions of the intervertebral foramen, concluding that patients with developmental central stenosis likely harbor concomitant foraminal stenosis [5]. Another lumbar study similarly reported that dural sac cross-sectional area significantly influenced foraminal area [16]. The absence of analogous strong correlations in the cervical spine may reflect regional anatomic differences, including the uncovertebral joints, cervical facet morphology, and the oblique orientation of the cervical neural foramina. Direct comparisons should be made cautiously because of differences in anatomy, imaging methodology, and cohort characteristics.

The lack of consistent findings for foraminal axial width and craniocaudal height is likely due in part to the inherent limitations of width and height in fully capturing the three-dimensional nature of the neural foramen. These linear measurements may incompletely capture the complex morphology of the neural foramen, particularly given the oblique orientation of the cervical foramina. Width as measured on the axial view reflects a single anteroposterior dimension at one slice level, and craniocaudal height similarly represents a single sagittal measurement. Volumetric measurement approaches or oblique sagittal reconstructions aligned to individual nerve root trajectories may better characterize foraminal morphology and could yield different correlation patterns; future studies employing such methods would be valuable [17,18].

The primary implication of these findings is quantitative: central canal dimensions should not be used as a surrogate for neural foraminal dimensions in morphometric research or quantitative imaging analysis. Although central canal stenosis and foraminal stenosis are already assessed as clinically distinct findings in routine practice, quantitative morphometric studies, including those developing compartment-specific reference values, automated segmentation tools, or imaging-based phenotypes, should treat these structures as distinct measurements rather than interchangeable proxies. Within this cohort, a larger central canal measurement did not reliably indicate larger foraminal dimensions, and vice versa; morphometric models that assume proportional scaling between compartments may therefore be systematically inaccurate.

Understanding the relationship between the cervical central canal and neural foramina may also be relevant in the management of patients with congenital cervical stenosis. Had this study detected a definite size relationship between the bony anatomy of the central canal and neural foramina, it might have been reasonable to suspect concomitant neural foraminal stenosis in individuals already diagnosed with congenital cervical stenosis. However, we did not detect any such association. While we did detect a modest-to-moderate correlation between IPD and neural foraminal area, the pathoanatomic changes associated with congenital cervical stenosis primarily involve the posterior elements and their effect on sagittal canal diameter, suggesting IPD may be less directly implicated in the narrowing mechanism [6,15]. As such, central canal stenosis and neural foraminal stenosis should be evaluated separately, even in patients with known congenital anomalies.

These data may also serve as a young adult reference baseline for future comparative studies examining how the canal-foramen relationship changes in older, degenerative, or symptomatic populations, including patients with cervical myelopathy, radiculopathy, or congenital stenosis. Such studies would help clarify whether degenerative processes, including disc collapse, uncovertebral hypertrophy, and facet arthropathy, affect each compartment proportionally or differentially.

This study has several limitations. Although patients were systematically screened to exclude apparent cervical spinal pathology, the cohort was derived from clinically indicated CT imaging rather than healthy-volunteer imaging; therefore, the possibility of selection bias related to imaging indication cannot be excluded, and the cohort should not be interpreted as a truly normative reference population. The retrospective, single-center design may also limit generalizability across institutions and patient populations. CT imaging characterizes osseous anatomy and does not account for soft-tissue contributors to stenosis, including disc protrusion, ligamentum flavum hypertrophy, or synovial cysts, which become particularly relevant in degenerative populations [19,20]. Detailed CT acquisition and reconstruction parameters, including slice thickness, reconstruction interval, and reconstruction kernel, were not uniformly available across the retrospective imaging period and could not be confirmed for all scans; measurement reproducibility across institutions with different CT protocols would require prospective evaluation. Although consistent measurement planes were applied, the oblique orientation and complex three-dimensional morphology of the cervical neural foramina may introduce measurement variability that linear measurements do not fully capture. This study was also limited to Pearson correlation analysis and did not include sex-stratified, body-size-adjusted, or multivariable analyses; residual confounding by demographic or anthropometric factors cannot be excluded. Finally, the absence of prospectively collected symptom data, standardized neurologic examination findings, or surgical outcomes limits direct clinical applicability. Future studies should evaluate these relationships in older, symptomatic, degenerative, and surgically treated populations.

## 5. Conclusions

In this large young adult CT cohort without apparent cervical spinal pathology, cervical central canal and neural foraminal dimensions demonstrated no strong correlations across levels. Although the neural foraminal area showed modest-to-moderate associations with interpedicular distance from C2–C3 to C6–C7, most other canal-to-foramen relationships were weak or inconsistent. These findings suggest that central canal dimensions should not be used as a surrogate for neural foraminal dimensions in quantitative morphometric assessment and may provide a young adult reference baseline for future comparative studies in degenerative and symptomatic populations.

## Figures and Tables

**Table 1:** Mean Cervical Central Canal and Neural Foraminal Dimensions, by Disc Level.

Disc Level	Central Canal			Left Neural Foramina			Right Neural Foramina		
	APD	IPD	Area	Width	Height	Area	Width	Height	Area
*C2* *–C3*	15.2 ± 2.2	21.4 ± 3.9	281.0 ± 72.0	6.8 ± 1.8	9.4 ± 2.8	64.2 ± 23.8	6.9 ± 1.8	9.4 ± 3.4	64.0 ± 24.6
*C3* *–C4*	14.1 ± 1.7	21.8 ± 4.7	258.0 ± 47.0	6.3 ± 1.7	8.8 ± 3.8	54.8 ± 18.6	6.2 ± 1.6	8.7 ± 2.8	55.1 ± 19.5
*C4* *–C5*	14.3 ± 1.6	22.5 ± 5.0	266.0 ± 45.0	6.4 ± 1.5	9.1 ± 1.8	57.9 ± 19.4	6.4 ± 1.5	9.1 ± 3.2	58.5 ± 19.9
*C5* *–C6*	14.7 ± 1.9	22.6 ± 5.1	275.0 ± 49.0	6.4 ± 1.5	9.5 ± 3.2	59.6 ± 19.2	6.5 ± 1.5	9.6 ± 4.6	60.3 ± 20.0
*C6* *–C7*	15.2 ± 2.2	22.0 ± 4.9	273.0 ± 56.0	6.5 ± 1.6	9.6 ± 2.1	58.6 ± 19.1	6.6 ± 1.6	9.7 ± 2.9	60.7 ± 21.8
*C7* *–T1*	16.1 ± 2.3	—	256.0 ± 58.0	6.5 ± 1.7	9.7 ± 2.2	58.2 ± 20.9	6.6 ± 1.6	9.6 ± 2.1	58.3 ± 21.0

APD = anteroposterior diameter; IPD = interpedicular distance; — = not measured. Linear measurements are reported in millimeters (mm); area measurements are reported in square millimeters (mm^2^).

**Table 2:** Pearson Correlations Between Cervical Central Canal Dimensions and Neural Foraminal Area, by Disc Level.

Disc Level	Left FA vs. APD	Left FA vs. IPD	Left FA vs. Canal Area	Right FA vs. APD	Right FA vs. IPD	Right FA vs. Canal Area
*C2–C3*	**0.234**	**0.541**	**0.271**	**0.276**	**0.587**	**0.301**
*C3–C4*	0.053	**0.564**	**0.128**	**0.07**	**0.602**	**0.162**
*C4–C5*	0.056	**0.539**	**0.126**	−0.007	**0.579**	**0.087**
*C5–C6*	**0.096**	**0.515**	**0.177**	0.038	**0.535**	**0.146**
*C6–C7*	**0.122**	**0.504**	**0.206**	**0.114**	**0.553**	**0.209**
*C7–T1*	**0.213**	—	**0.286**	**0.221**	—	**0.324**

Bold values denote *p* < 0.05. APD = anteroposterior diameter; IPD = interpedicular distance; FA = foraminal area; — = not measured. Full correlation matrix is provided in Supplementary Table S1.

## Data Availability

The data presented in this study are available upon reasonable request from the corresponding author. The data are not publicly available due to patient privacy and institutional restrictions.

## Supplementary Materials

The following supporting information can be downloaded at https://www.mdpi.com/article/10.3390/tomography12060086/s1. Supplementary Table S1: Complete Pearson Correlation Matrix Among Cervical Central Canal and Neural Foraminal Dimensions, by Disc Level.

## Abbreviations

The following abbreviations are used in this manuscript:

AP	Anteroposterior
BMI	Body Mass Index
CI	Confidence Interval
CT	Computed Tomography
ICC	Intraclass Correlation Coefficient
IPD	Interpedicular Distance
NFD	Neural Foraminal Dimensions
SD	Standard Deviation
