# Supplementary material for: Relationship Between Cervical Central Canal and Neural Foraminal Dimensions in a Normative Population
Source: Tomography. 2026 Jun 12;12(6):86. doi: 10.3390/tomography12060086 (PMC13306614; doi:10.3390/tomography12060086)
Supplement: Supplementary file 1 [file tomography-12-00086-s001.zip › tomography-4307295-supplementary.pdf]

Supplementary Table S1. Complete Pearson Correlation Matrix Among Cervical Central Canal and Neural Foraminal Dimensions, by Disc Level.

**Measurement**			**Left NFD**			**Right NFD**			**Central Canal**		
			**Width**	**Height**	**Area**	**Width**	**Height**	**Area**	**APD**	**IPD**	**Area**
C2-C3	*Left NFD*	*Width*	1.000	**.132**	**.079**	**.592**	**.072**	**.075**	-.011	.034	-.027
		Height	**.132**	1.000	**.407**	.068	**.259**	**.349**	.019	**.243**	.05
		Area	**.079**	**.407**	1.000	-.01	**.303**	**.702**	**.234**	**.541**	**.271**
	Right NFD	*Width*	**.592**	.068	-.01	1.000	.051	.053	-.005	-.013	-.022
		Height	**.072**	**.259**	**.303**	.051	1.000	**.331**	.019	**.222**	.038
		Area	**.075**	**.349**	**.702**	.053	**.331**	1.000	**.276**	**.587**	**.301**
	Central Canal	*APD*	-.011	.019	**.234**	-.005	.019	**.276**	1.000	**.273**	**.659**
		IPD	.034	**.243**	**.541**	-.013	**.222**	**.587**	**.273**	1.000	**.317**
		Area	-.027	.05	**.271**	-.022	.038	**.301**	**.659**	**.317**	1.000
C3-C4	*Left NFD*	*Width*	1.000	-.034	.042	**.67**	.039	-.014	**.115**	-.021	**.096**
		Height	-.034	1.000	**.338**	-.055	**.122**	**.162**	-.045	**.101**	.001
		Area	.042	**.338**	1.000	-.008	**.238**	**.687**	.053	**.564**	**.128**
	Right NFD	*Width*	**.67**	-.055	-.008	1.000	**.077**	.034	**.095**	-.03	**.1**
		Height	.039	**.122**	**.238**	**.077**	1.000	**.389**	-.048	**.191**	-.002
		Area	-.014	**.162**	**.687**	.034	**.389**	1.000	**.07**	**.602**	**.162**
	Central Canal	*APD*	**.115**	-.045	.053	**.095**	-.048	**.07**	1.000	**.155**	**.771**
		IPD	-.021	**.101**	**.564**	-.03	**.191**	**.602**	**.155**	1.000	**.272**
		Area	**.096**	.001	**.128**	**.1**	-.002	**.162**	**.771**	**.272**	1.000
C4-C5	*Left NFD*	*Width*	1.000	**.134**	**.114**	**.619**	**.104**	.059	.06	.009	.026
		Height	**.134**	1.000	**.632**	**.097**	**.276**	**.439**	**-.127**	**.241**	**-.137**
		Area	**.114**	**.632**	1.000	.059	**.208**	**.678**	.056	**.539**	**.126**
	Right NFD	*Width*	**.619**	**.097**	.059	1.000	.054	.041	.039	-.041	.068
		Height	**.104**	**.276**	**.208**	.054	1.000	**.349**	-.02	**.19**	-.002
		Area	.059	**.439**	**.678**	.041	**.349**	1.000	-.007	**.579**	**.087**
	Central Canal	*APD*	.06	**-.127**	.056	.039	-.02	-.007	1.000	**.118**	**.768**
		IPD	.009	**.241**	**.539**	-.041	**.19**	**.579**	**.118**	1.000	**.248**
		Area	.026	**-.137**	**.126**	.068	-.002	**.087**	**.768**	**.248**	1.000
C5-C6	*Left NFD*	*Width*	1.000	**.093**	**.109**	**.645**	.054	.068	-.013	**-.078**	-.012
		Height	**.093**	1.000	**.278**	.05	**.118**	**.246**	-.052	**.099**	-.046
		Area	**.109**	**.278**	1.000	.063	**.163**	**.651**	**.096**	**.515**	**.177**
	Right NFD	*Width*	**.645**	.05	.063	1.000	.005	**.095**	.027	**-.098**	.042
		Height	.054	**.118**	**.163**	.005	1.000	**.249**	-.015	**.092**	-.022
		Area	.068	**.246**	**.651**	**.095**	**.249**	1.000	.038	**.535**	**.146**

	Central Canal	*APD*	-.013	-.052	**.096**	.027	-.015	.038	1.000	**.18**	**.781**
		IPD	**-.078**	**.099**	**.515**	**-.098**	**.092**	**.535**	**.18**	1.000	**.327**
		Area	-.012	-.046	**.177**	.042	-.022	**.146**	**.781**	**.327**	1.000
C6-C7	*Left NFD*	*Width*	1.000	**.198**	**.186**	**.657**	.056	**.076**	.023	-.016	-.003
		Height	**.198**	1.000	**.674**	**.095**	**.317**	**.325**	-.035	**.216**	.01
		Area	**.186**	**.674**	1.000	.063	**.336**	**.566**	**.122**	**.504**	**.206**
	Right NFD	*Width*	**.657**	**.095**	.063	1.000	.06	**.095**	-.034	-.035	-.063
		Height	.056	**.317**	**.336**	.06	1.000	**.427**	-.05	**.217**	.02
		Area	**.076**	**.325**	**.566**	**.095**	**.427**	1.000	**.114**	**.553**	**.209**
	Central Canal	*APD*	.023	-.035	**.122**	-.034	-.05	**.114**	1.000	**.245**	**.815**
		IPD	-.016	**.216**	**.504**	-.035	**.217**	**.553**	**.245**	1.000	**.374**
		Area	-.003	.01	**.206**	-.063	.02	**.209**	**.815**	**.374**	1.000
C7-T1	*Left NFD*	*Width*	1.000	**.22**	**.272**	**.694**	**.176**	**.253**	**.117**	-	**.116**
		Height	**.22**	1.000	**.701**	**.144**	**.394**	**.375**	**.084**	-	**.175**
		Area	**.272**	**.701**	1.000	**.181**	**.362**	**.54**	**.213**	-	**.286**
	Right NFD	*Width*	**.694**	**.144**	**.181**	1.000	**.169**	**.267**	**.148**	-	**.142**
		Height	**.176**	**.394**	**.362**	**.169**	1.000	**.699**	**.117**	-	**.209**
		Area	**.253**	**.375**	**.54**	**.267**	**.699**	1.000	**.221**	-	**.324**
	Central Canal	*APD*	**.117**	**.084**	**.213**	**.148**	**.117**	**.221**	1.000	-	**.819**
		IPD	-	-	-	-	-	-	-	-	-
		Area	**.116**	**.175**	**.286**	**.142**	**.209**	**.324**	**.819**	-	1.000

[1]APD = anteroposterior diameter; IPD = interpedicular distance; NFD = neural foraminal dimensions; — = not measured. Bold values denote p < .05.
